# Modern Pathologic Diagnosis of Renal Oncocytoma

**DOI:** 10.15586/jkcvhl.2017.96

**Published:** 2017-10-09

**Authors:** Sara E. Wobker, Sean R. Williamson

**Affiliations:** 1 Department of Pathology and Laboratory Medicine, University of North Carolina at Chapel Hill, Chapel Hill, NC, USA; 2 Lineberger Comprehensive Cancer Center, Chapel Hill, NC, USA; 3 Department of Pathology and Laboratory Medicine and Henry Ford Cancer Institute, Henry Ford Health System, Detroit, MI, USA; 4 Department of Pathology, Wayne State University School of Medicine, Detroit, MI, USA

**Keywords:** chromophobe renal cell carcinoma, kidney, oncocytic neoplasm, oncocytoma, renal mass, renal mass biopsy, succinate dehydrogenase-deficient renal cell carcinoma

## Abstract

Oncocytoma is a well-defined benign renal tumor, with classic gross and histologic features, including a tan or mahogany-colored mass with central scar, microscopic nested architecture, bland cytology, and round, regular nuclei with prominent central nucleoli. As a result of variations in this classic appearance, difficulty in standardizing diagnostic criteria, and entities that mimic oncocytoma, such as eosinophilic variant chromophobe renal cell carcinoma and succinate dehydrogenase-deficient renal cell carcinoma, pathologic diagnosis remains a challenge. This review addresses the current state of pathologic diagnosis of oncocytoma, with emphasis on modern diagnostic markers, areas of controversy, and emerging techniques for less invasive diagnosis, including renal mass biopsy and advanced imaging.

## Introduction

Oncocytoma has been recognized for decades (1, 2) as a distinct subtype of benign renal tumor; however, despite evaluation of numerous biomarkers performed over the years (3, 4), pathologic diagnosis of oncocytoma and distinction from its mimics remain a challenge, even today (5). This review addresses the current state of pathologic diagnosis of oncocytoma, with emphasis on modern diagnostic markers, areas of controversy, and emerging techniques for less invasive diagnosis, including renal mass biopsy and specialized imaging techniques.

## Gross Pathology

**Figure 1:**
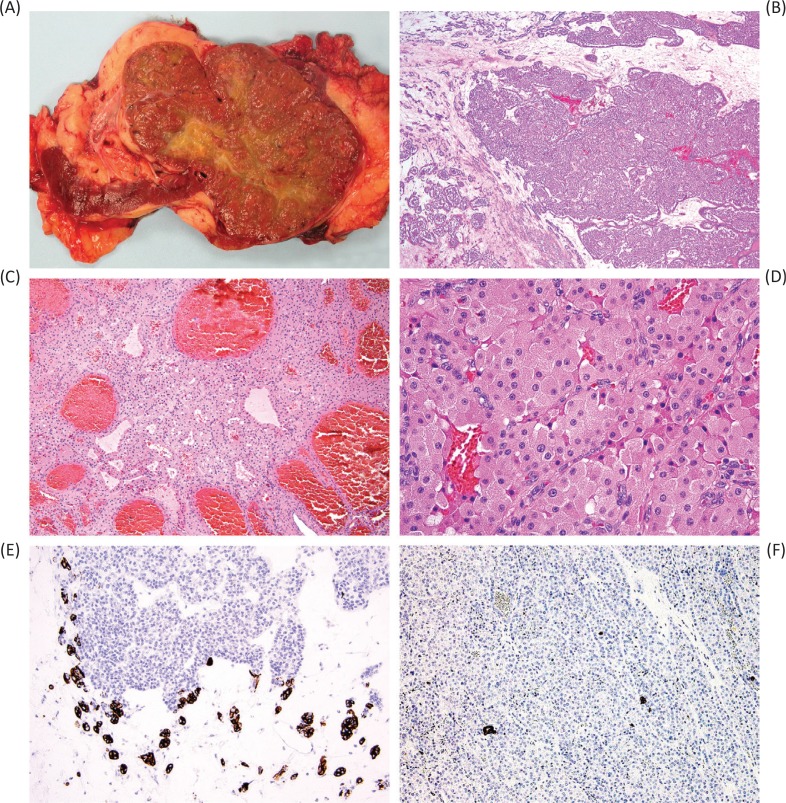
The characteristic gross appearance of oncocytoma (A) varies depending on the size of the tumor, but often has a similar color to normal renal parenchyma (mahogany brown) and characteristically, but not consistently, a central scar. Microscopic appearance typically includes nests dispersed in fibrous stroma (B) and can include tubular structures (C) or densely packed nests (D). Immunohistochemical staining for CK7 can be increased in the central scar area (E) but is typically limited to only scattered cells and small clusters of cells in other areas (F).

The characteristic gross appearance of oncocytoma includes a tan or mahogany brown cut surface (2, 6–8), generally similar to normal renal parenchyma in color and in contrast to the golden yellow cut surface of clear cell renal cell carcinoma. Although a central scar is quite characteristic of oncocytoma (Figure 1A), it is not specific for oncocytoma and is not present in all tumors (2, 6–8). A central scar can also be found in chromophobe renal cell carcinoma, as well as other slow growing neoplasms, and substantial hyalinization and fibrosis can also be present within clear cell renal cell carcinoma. With the increasing identification of renal masses incidentally via imaging techniques, the size of oncocytoma tumors can also range from small solid nodules without central scar to large masses that would otherwise be concerning for high-stage renal cell carcinoma.

## Histopathology

The classic histologic appearance of renal oncocytoma includes nests and tubular structures lined by cells with eosinophilic, granular cytoplasm (Figure 1B–D). Uncertainty in the diagnosis can arise when other patterns are present, such as a highly compact nested architecture, resulting in an almost entirely solid appearance, or when small papillary structures protrude into cystic spaces, raising concern of an eosinophilic variant of papillary renal cell carcinoma. Oncocytomas typically contain edematous myxoid or hyalinized stroma, often resulting in at least some areas with nests and tubular structures dispersed in this stroma. Clear cytoplasm may also be focally present, typically in the area of the central scar.

Nuclei are characteristically round and regular; however, it is also established that oncocytomas can contain areas of “degenerative” cytologic atypia, resulting in patches of tumor cells with large nuclei, irregular nuclear contours, and smudged chromatin (8). Similar to other pseudomalignant features discussed elsewhere in this review, this finding is generally considered compatible with a benign diagnosis (8), especially if other worrisome features such as increased mitotic activity are lacking. Mitotic activity is typically extremely rare in oncocytoma, and one of the most agreed-upon features is that finding a single mitotic figure with careful search may still be compatible with the diagnosis. However, more than one readily identifiable mitotic figure is quite worrisome or potentially incompatible with a diagnosis of oncocytoma (5).

An unusual pattern that is sometimes encountered in oncocytoma is the finding of areas with more scant cytoplasm, resulting in an appearance variably referred to as “small cell” oncocytoma, oncocytoma with pseudorosettes, or “oncoblastic” cells (9). In general, such tumors appear to have overall immunohistochemical and molecular features similar to usual oncocytomas, at least regarding the most widely employed markers (9).

## Immunohistochemistry

In considering the differential diagnosis of renal oncocytoma and other oncocytic neoplasms, the use of immunohistochemistry and special stains can be instructive. Oncocytomas generally show very minimal staining for cytokeratin 7 (CK7), typically limited to scattered individual cells or small clusters of cells (5, 10), whereas a classic example of chromophobe renal cell carcinoma (with pale-staining cytoplasm) is diffusely positive in a membranous distribution. In a recent study of urologic pathologists, CK7 was the most commonly utilized staining technique for diagnosing oncocytoma, although a specific threshold of positive staining incompatible with oncocytoma was not well agreed upon (5). However, eosinophilic examples of chromophobe renal cell carcinoma may exhibit less extensive CK7 labeling (11), making selection of a positive staining threshold more challenging. In the survey of urologic pathologists, there was greatest acceptance for <5% of cells staining positively as supportive of oncocytoma, whereas comfort with an oncocytoma diagnosis decreased for greater extents of staining and entirely negative staining (5).

A confusing aspect of the existing published literature on CK7 staining in oncocytoma is that there is considerable variability between reported “positive” and “negative” results (3, 4), probably resulting from selection of cutoff thresholds, in which some authors have interpreted minimal staining of <5% of cells as negative and others have interpreted this as positive. In addition, the central scar area of oncocytoma may exhibit increased staining for CK7 (Figure 1E) compared to the areas with usual morphology (Figure 1F), which could also potentially influence published results and lead to diagnostic challenges for anyone unfamiliar with this phenomenon (12). The International Society of Urological Pathology (ISUP) consensus for best practices in immunohistochemistry recommends that immunohistochemistry be used in this context only for borderline cases (predominantly placing diagnostic emphasis on morphology and growth pattern) and notes CK7 as the most helpful marker in this scenario (11).

Other markers, such as kidney-specific cadherin and S100A1, are used by some laboratories, although these are less widely employed (5, 13–15). The use of colloidal iron staining (Hale or modified Mowry) is also often invoked in this setting as a histochemical technique. However, variation in staining techniques between laboratories can make interpretation challenging, leading to variable use across practices. When working properly, oncocytomas will show negative or luminal staining, whereas chromophobe renal cell carcinomas will show diffuse, reticular cytoplasmic staining (16).

For distinction of oncocytoma and chromophobe renal cell carcinoma from other renal cell carcinoma subtypes, such as clear cell and papillary renal cell carcinoma variants with eosinophilic cytoplasm, markers such as KIT (CD117) and vimentin may be helpful, as both oncocytoma and chromophobe renal cell carcinoma share frequent membranous positivity for KIT and negative staining for vimentin, in contrast to clear cell and papillary renal cell carcinoma, which characteristically have the opposite findings (negative KIT and often positive vimentin) (11, 17). Of note, vimentin staining is also commonly positive in the areas of central scar in oncocytoma, similar to the increased staining for CK7 in these areas, which may be a diagnostic pitfall, as it differs from the expected results (18).

## Local Invasion

**Figure 2:**
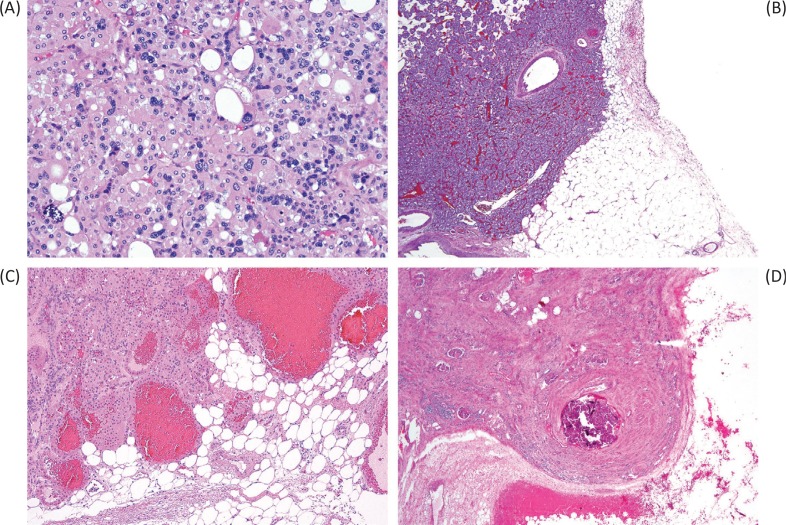
Pseudomalignant features in oncocytoma can include degenerative-type atypia (A), intermingling of the tumor with fat (B–C), and vascular invasion or large vein invasion (D). Despite that these would confer a high stage to renal cell carcinoma, they are currently thought to have no impact on the benign behavior of oncocytoma.

In addition to the pseudomalignant finding of degenerative type atypia, as discussed previously (Figure 2A), it is relatively well known and accepted that oncocytomas can “invade” or interdigitate with fat (Figure 2B–C), particularly perinephric fat, and to date this finding has not appeared to alter the benign clinical behavior of these tumors (6–8, 19). A potential explanation for this tendency to interdigitate with fat is that oncocytomas (as well as chromophobe renal cell carcinomas) tend to have a less continuous tumor pseudocapsule when compared to other renal cell carcinoma types, such as clear cell and papillary renal cell carcinoma (20). Although this is conceptually similar, a recent study of urologic pathologists revealed greater uncertainty for involvement of the renal sinus fat, in contrast to perinephric fat, the latter being more readily accepted as compatible with a benign diagnosis (5). Our interpretation is that involvement of fat in either of these locations remains compatible with an oncocytoma diagnosis. Notably, this invasion or interdigitation typically does not elicit any stromal reaction or response and has a scalloped or ballooning contour.

It is also interesting that several reports have encountered vein or vein branch invasion in renal oncocytoma (Figure 2D), again noting that this does not appear to affect the benign behavior of oncocytoma (8, 21, 22). In renal cell carcinomas, extension into a vein or vein branch would warrant a pT3a pathologic stage category; however, vascular involvement by benign renal tumors, especially angiomyolipoma and rarely other entities, has also been reported (23–25). These studies suggest that vascular involvement is not inherently indicative of malignancy alone and may occur particularly in renal tumors, through poorly understood mechanisms. Vascular extension by benign tumors of other organs has also been described, most notably intravascular leiomyomatosis of the gynecologic tract (26). This makes the distinction of oncocytoma from renal cell carcinoma even more critical, as it involves discriminating a benign neoplasm with vascular extension from a high-stage renal cell carcinoma, the latter being potentially eligible for enrollment in clinical trials for adjuvant treatment.

## Genetics

Many advanced genetic studies of renal oncocytoma have been employed, largely in the research setting. However, traditional cytogenetics (karyotyping), comparative genomic hybridization (CGH), and fluorescence *in situ* hybridization (FISH) are sometimes utilized in clinical diagnostic work (5). Based on karyotype data, oncocytomas have been generally found to exhibit either a diploid karyotype, loss of chromosome 1, loss of Y, or rearrangement of 11q13, including t(5;11)(q35;q13) (2). As it is the locus of the *CCND1* gene (cyclin D1), it has been subsequently found that many of these likely represent rearrangements involving *CCND1*, and emerging data suggest that oncocytomas harboring such rearrangements may represent a distinctive tumor subset (27, 28).

Sukov et al. found that more than half of oncocytomas with cyclin D1 immunohistochemical labeling had *CCND1* rearrangement by FISH, compared to only a single tumor with rearrangement by FISH but without cyclin D1 staining (27). Interestingly, most of the cyclin D1-positive oncocytomas were solitary, whereas there was a considerable rate of multifocality in the cyclin D1-negative patients (27). In another study, Joshi et al. proposed dividing oncocytomas into type 1 and type 2 tumors, the former characterized by a diploid karyotype and *CCND1* rearrangement. Conversely, the type 2 group was associated with recurrent loss of chromosome 1, X, Y, 14, or 21, arguing for more genetic overlap with chromophobe renal cell carcinoma in this subgroup and leading the authors to speculate that this group might represent a precursor to eosinophilic variant chromophobe renal cell carcinoma (28).

Both oncocytoma and chromophobe renal cell carcinoma are noted to have mutations in mitochondrial genes (28–30). However, chromophobe renal cell carcinoma characteristically exhibits multiple chromosomal losses, including commonly chromosomes Y, 1, 2, 6, 10, 13, 17, and 21 (29) and lesser rates of chromosomes 3, 5, 8, 9, 11, and 18 (30). Other authors have also found chromosomal gains (31). *TP53* appears to be among the more commonly mutated genes in chromophobe renal cell carcinoma, followed by *PTEN* (30). Additionally, *TERT* gene promoter rearrangements have also been found to occur in a subset of chromophobe tumors (30). Although some authors have found eosinophilic variant chromophobe renal cell carcinoma to harbor a similar pattern of chromosomal loss to usual chromophobe tumors when defined strictly (32), other studies have found a lower rate of chromosomal abnormality in eosinophilic variant chromophobe tumors, further blurring the distinction between these two diagnostic entities (30).

In some unique contexts, such as the Birt–Hogg–Dubé syndrome and renal oncocytosis (multiple oncocytic renal tumors without findings of Birt–Hogg–Dubé syndrome) (33), tumors are often referred to as oncocytomas, chromophobe renal cell carcinomas, or hybrid oncocytoma–chromophobe tumors (HOCT) (34, 35). However, it remains incompletely understood whether these should be regarded as true oncocytomas and chromophobe tumors, or whether they should be considered pathologically and genetically distinct entities (36). Some authors have found that intermediate stages of chromosomal alterations can be found in this setting, suggesting a stepwise progression from oncocytoma to chromophobe renal cell carcinoma (33), whereas others have found neoplasms of oncocytosis to have some differences in chromosomal aberration patterns, compared to usual oncocytoma and chromophobe renal cell carcinoma, suggesting that these neoplasms are molecularly distinct (34). “Hybrid tumors” have also been reported sporadically (without the context of a multiple tumor syndrome), which appear to be associated with multiple chromosomal gains and losses (37).

Overall, knowledge of these typical chromosomal alterations can be helpful in assessing challenging cases, including resection specimens, whether by conventional karyotyping, FISH, and CGH, or through other methods. In general, a diploid karyotype or loss of chromosome 1 in the appropriate morphologic and immunohistochemical context can be considered supportive of an oncocytoma diagnosis, whereas other losses or other alterations not typical of oncocytoma might be used to favor a borderline diagnosis or classification as chromophobe renal cell carcinoma.

## Renal Mass Biopsy

**Figure 3:**
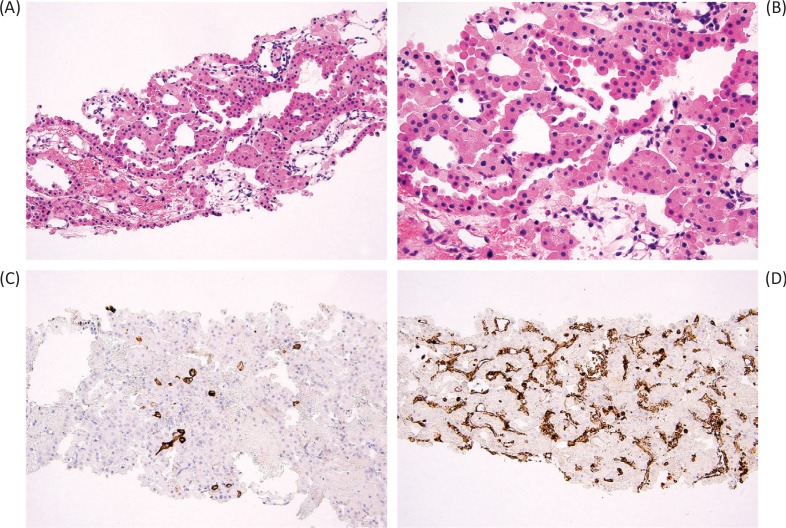
Core needle biopsy of oncocytoma (A) demonstrates tubular structures lined by homogeneous eosinophilic cells with round, regular nuclei (B). The typical immunohistochemical staining pattern includes rare CK7-positive cells (C) and negative vimentin staining (D), the latter highlighting only the vasculature.

Core needle biopsies are increasingly used in the diagnosis of renal masses (Figure 3). Because 20–45% of small renal masses are ultimately found to be benign, active surveillance is an option for many patients (38, 39). The diagnostic accuracy of renal mass biopsy remains somewhat controversial, however. Individual groups have reported up to 80% diagnostic rate from renal mass biopsy, with the ability to provide subtype and nuclear grade in the majority of diagnostic biopsies (40).

Unfortunately, oncocytic lesions can be especially troublesome in renal mass biopsy, as interpreting only a limited sampling of tumor may not be representative of the entire lesion. A meta-analysis of 205 oncocytic renal mass biopsies from 2017 showed that the positive predictive value for a diagnosis of oncocytoma on renal mass biopsy was 67% with significant heterogeneity and wide confidence interval, indicating that the diagnostic accuracy varies greatly between studies and, by extrapolation, between pathologists (41).

For renal mass biopsies of oncocytic neoplasms, there is a split among urologic pathologists as to whether it is preferable to issue an outright diagnosis of oncocytoma (when features are typical in the biopsy sample) or to use more general terminology, such as “oncocytic neoplasm,” with comment that the features are compatible with oncocytoma (5). In the context that morphologic and immunohistochemical features are largely compatible with oncocytoma, yet in which there are minor equivocal features, such as variation in cell size or slight nuclear irregularity, it is also reasonable to utilize a borderline diagnostic category expressing uncertainty between oncocytoma and eosinophilic variant chromophobe renal cell carcinoma. In this setting, immunohistochemical staining may also be helpful. Although CK7 staining may not be beyond the expected level of oncocytoma, findings such as negative vimentin staining and positive KIT staining generally argue against other considerations, such as papillary or clear cell renal cell carcinoma with eosinophilic cells. Since chromophobe renal cell carcinoma, especially the eosinophilic variant, is also generally regarded as a less aggressive tumor subtype, this can facilitate appropriate management in patients who are candidates for nonsurgical treatment (42). Conversely, if nuclear or cytologic features are inconsistent with oncocytoma (non-degenerative atypia, nuclear membrane irregularity, or perinuclear clearing), a diagnosis of eosinophilic variant of chromophobe renal cell carcinoma may be favored.

## Differential Diagnosis

Although oncocytic or eosinophilic areas can be seen in almost any renal neoplasm, there are a few entities which may closely resemble oncocytoma throughout the majority of the mass. Broadly, these are neoplasms with a solid or nested pattern of growth and abundant eosinophilic cytoplasm. A few entities most likely to mimic oncocytoma are discussed below (Table 1).

**Table 1:** Typical characteristics of oncocytoma and differential diagnostic considerations

	Central scar	Papillary structures	Classic architectural pattern	Nuclei	CK7	KIT	Vimentin	AMACR	SDHB	Melanocytic markers
**Oncocytoma**	Classic but only subset	No or rare abortive	Nested, tubules	Round, regular with central nucleoli	Rare single cells and clusters of cells	+	- (except central scar)	Variable but usually lesser intensity	+	-
**Chromophobe RCC, eosinophilic**	Sometimes	No	Solid, trabecular	Irregular, wrinkled (variable)	Variable to diffuse	+	-	Variable but usually lesser intensity	+	-
**Chromophobe RCC, classic**	Sometimes	No	Solid, trabecular	Irregular, wrinkled	+ (diffuse)	+	-	Variable but usually lesser intensity	+	-
**SDH-deficient RCC**	Unknown	Usually no (rare variant cases)	Solid	Round, regular	-	- (mast cells present)	- (usually)	Variable but usually lesser intensity	- (loss)	-
**Papillary RCC, eosinophilic**	Usually not	Yes	Papillary	Usually round to oval, regular	Variable, sometimes minimal	-	+	Strong	+	-
**Tubulocystic RCC**	Usually not	No	Cystic	Hobnailing, usually round with macronucleoli	Variable	-	+	Strong	+	-
**Epithelioid AML**	Usually not	No	Solid	Round, oval, prominent nucleoli, variable mild to marked atypia, multinucleate forms	-	-	+	No data	+	+

*Note*: Most common findings are reported. RCC, renal cell carcinoma; SDH, succinate dehydrogenase; AML, angiomyolipoma; AMACR, alpha-methyl-acyl-coA racemase; SDHB, succinate dehydrogenase B.

### Chromophobe renal cell carcinoma

Chromophobe renal cell carcinoma has the most morphologic overlap with oncocytoma, particularly the eosinophilic variant, and presents the main diagnostic conundrum. Indeed, since there are “gray zone” tumors that are difficult to classify as either oncocytoma or eosinophilic variant of chromophobe renal cell carcinoma (8), it is tempting to consider these diagnoses as a spectrum. Grossly, chromophobe renal cell carcinoma can also mimic oncocytoma, presenting as a well-circumscribed tan-brown mass, sometimes also containing a central scar. The classic histologic appearance of chromophobe renal cell carcinoma includes solid or trabecular architecture, some nuclear atypia, well-preserved chromatin and wrinkled (“raisinoid”) nuclei, or more subtly occasional notched nuclei. Cells are often so voluminous, with low nuclear–cytoplasmic ratio, that some appear to have no nucleus at all due to sectioning artifact. Some tumors have substantial trabecular growth such that one could trace a long, imaginary line through tumor cells without crossing stroma or vasculature, something that would be impossible with the intricate vascular network of clear cell renal cell carcinoma or the nested architecture of oncocytoma.

**Figure 4:**
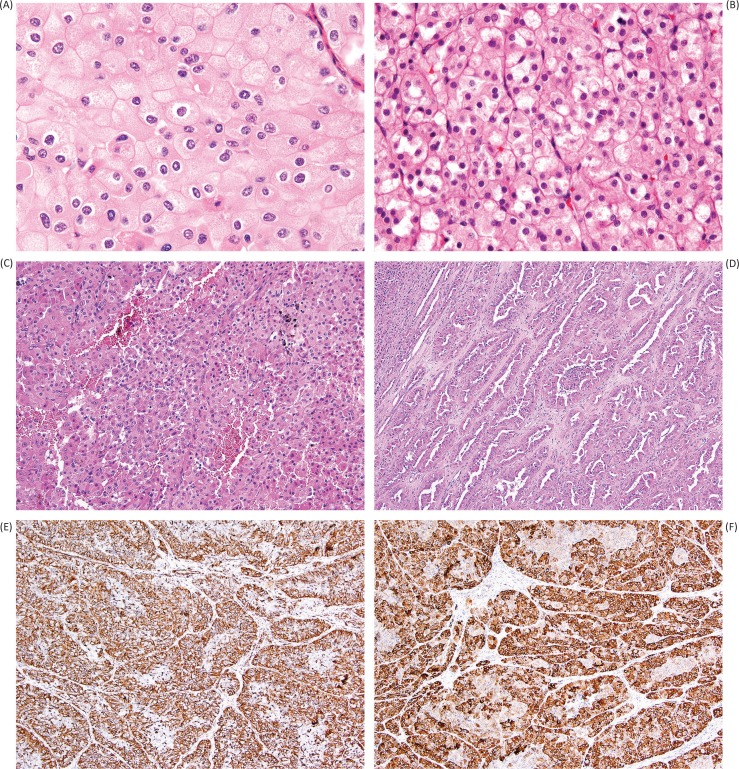
Mimics of oncocytoma include eosinophilic variant chromophobe renal cell carcinoma (A), in which distinction from oncocytoma is challenging, but is favored by perinuclear clearing (“halo”), nuclear wrinkling and irregularity, and substantial trabecular growth pattern. Succinate dehydrogenase-deficient renal cell carcinoma (B) is a more recently recognized entity that may closely mimic oncocytoma due to bland, uniform cytology; however, clues to this diagnosis include diffuse growth pattern (rather than tubular or nested formation) and cytoplasmic inclusions of flocculent material (sometimes referred to as vacuoles), likely representing large abnormal mitochondria. Papillary renal cell carcinoma with oncocytic features (C) can also mimic oncocytoma; however, this diagnosis should be suspected in the presence of substantive papillary formation (D) or positivity for vimentin (E). Labeling for alpha-methyl-acyl-coA racemase (AMACR) is typically very strong and diffuse in all patterns of papillary renal cell carcinoma (F).

The degree of nuclear membrane irregularity typically seen in chromophobe renal cell carcinoma is generally considered not compatible with the diagnosis of oncocytoma (Figure 4A); however, in the study of 109 oncocytomas by Trpkov et al., focal chromophobe renal cell carcinoma-like cytology (<5% of the neoplasm) did not alter the benign behavior of any tumor in the study (8). Other hallmark features include perinuclear cytoplasmic clearing or “halo,” as well as binucleate cells and well-defined cellular borders (the so-called vegetable cells) (43). As noted previously, although the ISUP recommendation is to rely predominantly on morphologic features for the differential diagnosis of oncocytoma versus chromophobe renal cell carcinoma, immunohistochemistry for CK7 can be useful in this setting, with more extensive labeling found in chromophobe renal cell carcinoma compared to the scattered individual cell pattern of oncocytoma. We would generally consider contiguous patches of positive CK7 labeling to argue against a diagnosis of oncocytoma, although a specific threshold of positive staining is not entirely agreed upon (5, 11, 12).

### Hybrid oncocytoma–chromophobe tumor

The term “hybrid oncocytoma–chromophobe tumor” is relatively widely used in the literature for tumors with mixed features of oncocytoma and chromophobe renal cell carcinoma. However, the definitive criteria for use of this terminology remain somewhat poorly defined. In general, this refers to a neoplasm with some areas resembling oncocytoma and other areas resembling chromophobe renal cell carcinoma, in our usage typically forming a mosaic or mixed pattern. However, reported usage of this term is variable, with some pathologists using it in the context of an apparent syndrome (multiple tumors), others using it only with mosaic or mixed morphology, and some others using it for any tumor with borderline features between oncocytoma and chromophobe renal cell carcinoma (5). Therefore, reported series of renal mass biopsies in which some oncocytomas were upgraded to malignancy as “hybrid tumor” at surgical resection may be confounded by the remaining uncertainty as to what exactly constitutes a true “hybrid.” Some of these may represent oncocytomas with borderline atypical features (8). At the ISUP Vancouver Classification of Renal Neoplasia, there was not agreement as to whether “hybrid tumor” represents a unique entity, and therefore it remained, for lack of consensus, considered as a subdivision of chromophobe renal cell carcinoma (36), an approach also employed by the 2016 World Health Organization Classification (29).

### Succinate dehydrogenase-deficient renal cell carcinoma

Succinate dehydrogenase (SDH)-deficient renal cell carcinoma is a recently recognized and rare entity that can also mimic oncocytoma due to its composition by cytologically bland, monomorphic, eosinophilic cells (Figure 4B) (44–47). These tumors are associated with an autosomal dominant mutation in subunits of the SDH complex genes, a component of the mitochondrial complex II. As part of the hereditary paraganglioma-pheochromocytoma syndrome, these tumors can be associated with other neoplasms, including gastrointestinal stromal tumor (GIST), paraganglioma, and pituitary adenoma (44–47).

Morphologically, SDH-deficient renal cell carcinoma is composed of sheets and nests of cells with clear to eosinophilic cytoplasm and low-grade nuclear features. Although the tumor cells may form glandular structures or cysts, they are often arranged in a rather diffuse pattern, which may leave doubt regarding the renal tubular nature (47). The classic histologic finding for these tumors is the presence of eosinophilic, flocculent inclusions within the cytoplasm (Figure 4B). Although sometimes referred to as cytoplasmic vacuoles, these structures likely represent enlarged, abnormal mitochondria (47, 48).

Since all normal cells should have a functioning Krebs cycle, and since detection of mitochondrial proteins is readily achieved in eosinophilic renal cell tumors (due to high mitochondrial density), the diagnosis of SDH-deficient renal cell carcinoma can be readily confirmed with abnormal negative immunohistochemical staining for the SDHB protein. Although rare renal tumors with mutations in other subunits of the SDH complex (49, 50) or negative SDHA staining (47) have been reported, in general it appears that defects in any of the subunits destabilize the enzyme complex, resulting in abnormal negative SDHB staining regardless of which gene harbors the mutation (51), making SDHB a useful screening immunohistochemical marker. Other immunohistochemical clues to this diagnosis include a complete lack of CK7 reactivity, contrasting to the rare scattered cells of oncocytoma, a paucity of positivity for epithelial markers overall, and negative staining for KIT (but often many intratumoral mast cells highlighted by KIT staining) (47).

### Papillary renal cell carcinoma with eosinophilic cells

Oncocytomas do not show papillary growth, with the exception of rare abortive tufts protruding into dilated tubules or microcysts (6–8); therefore, the identification of any areas with substantive papillary features will usually exclude the diagnosis of oncocytoma. Classification as an eosinophilic form of papillary renal cell carcinoma (Figure 4C–D) can be additionally supported by the presence of foamy macrophages, or by immunohistochemical staining results, such as positive vimentin (Figure 4E), very diffuse and strong labeling for alpha-methyl-acyl-coA racemase (AMACR, Figure 4F), and negative staining for KIT. Although positive CK7 is generally considered a feature of papillary renal cell carcinoma, this is less true in eosinophilic examples, and therefore limited CK7 staining may not be helpful in excluding the possibility of papillary renal cell carcinoma (52). Oncocytomas may have degenerative atypia, but frank atypia without the typical smudged chromatin pattern would also make this diagnosis unlikely. Clear cytoplasm may occasionally be seen in areas near the central scar of an oncocytoma, but predominantly clear cell features are not consistent with the diagnosis.

### Epithelioid angiomyolipoma

Epithelioid angiomyolipoma (or perivascular epithelioid cell neoplasm/PEComa) (53–55) can mimic oncocytoma as a solid or nested neoplasm with abundant eosinophilic cytoplasms. Although the trifecta of epithelioid eosinophilic cells, thick-walled blood vessels, and fat is diagnostic of the entity, occasionally the other components do not make up a significant proportion of the tumor (56–59). In the epithelioid variant of angiomyolipoma, nuclear atypia is usually quite marked, excluding a diagnosis of oncocytoma and raising consideration of a high-grade renal cell carcinoma. Large multinucleated cells are often present, sometimes containing central eosinophilic cytoplasm, a ring of nuclei, and peripheral clearing, somewhat reminiscent of Touton-type giant cells, as well as other multinucleate forms that have been variably described as “spider cells,” ameboid, ganglion-like, or strap-like (56–59). A rare variant of angiomyolipoma reported by Martignoni et al., however, was noted to more closely resemble oncocytoma (60). If there is any question as to the exact nature of the epithelioid cells, a PAX8 immunohistochemical stain can be used to identify renal epithelial cell lineage. Epithelioid angiomyolipoma will exhibit negative staining, whereas oncocytomas and other neoplasms of renal cell origin typically show diffuse nuclear positivity. Conversely, epithelioid angiomyolipoma will exhibit at least focal labeling for melanocytic markers, including HMB-45, melan-A, and MiTF, as well as usually diffuse labeling for cathepsin K (61).

### Tubulocystic renal cell carcinoma

Most cases of tubulocystic renal cell carcinoma would not be confused with oncocytoma due to their characteristic morphology of tubular and cystic spaces, lined by cells with eosinophilic cytoplasm and hobnail-shaped cells with prominent nucleoli. However, a recent study focused on examples of cystic oncocytoma, which may be a source of confusion, and compared them to tubulocystic renal cell carcinoma (62). Skenderi et al. found that tubulocystic renal cell carcinomas were more frequently positive for vimentin, AMACR, CD10, and CK7, and had a higher Ki67 proliferative index than cystic oncocytoma (62). Cystic oncocytomas were typically positive for KIT, in contrast to negative staining in tubulocystic renal cell carcinomas. Morphologically, islands of cells within the fibrous stroma and solid nests were also regarded as a helpful clue favoring oncocytoma over tubulocystic renal cell carcinoma (62).

## Imaging

Oncocytomas are usually asymptomatic and are often discovered incidentally on cross-sectional imaging performed for other indications. Much research is under way to determine a way to distinguish oncocytoma from renal cell carcinoma by imaging and, therefore, to potentially avoid surgery for a benign tumor (63–65). Although frequently associated with a central scar and hypervascularity, these findings are not sufficient to make a definitive diagnosis of oncocytoma (66). With the increased use of cross-sectional imaging, renal masses are being identified much more frequently with concomitant overtreatment of benign neoplasms, such as oncocytoma (67). Currently, there are no precise radiologic criteria for oncocytoma.

Recent work has shown promising results for the use of technetium-99m (^99m^Tc)-sestamibi single-photon emission computed tomography/x-ray computed tomography (SPECT/CT) to differentiate oncocytomas and hybrid oncocytic/chromophobe tumors from other renal cell carcinomas (68). In an updated series of cases, this new modality showed an overall sensitivity of 87.5% and a specificity of 95.2% (69).

## Conclusion

Oncocytomas are a common, benign renal neoplasm that may be detected incidentally. It is critical to identify them as benign and to recognize the potential pitfalls that may cause doubt about the diagnosis—renal fat involvement, vascular invasion, and degenerative atypia. With the increasing use of renal mass biopsy, it is also important to know the limitations of the technique and use appropriate criteria before diagnosing an oncocytoma in a limited biopsy specimen.

## Conflict of Interest

The authors declare no potential conflicts of interest with respect to research, authorship, and/or publication of this article.
